# The Expansion of Medicine

**Published:** 1890-08-23

**Authors:** 


### The Doctor's Pulpit.

### THE EXPANSION OF MEDICINE.

"To implant English ideas, English culture, English language, English institutions, and English rule in all the outlying places of the globe, that is, as I take it, the manifest destiny of our race." Thus writes Mr. Edward Dicey in the *Nineteenth Century*. We will first correct Mr. Dicey, and then agree with him. He means "British" when he says "English," and he ought to have written it so. The English form but a portion of the British people; the Scotch, the Irish, and the Welsh constitute other integral parts. It seems very clear and plain to us Britons that our language, our ideas, our culture, and our institutions, as well as ourselves, are destined to spread over the greater part of the globe. Most of us feel quite equal to the task of peopling, ruling, cultivating, and teaching this comparatively small sphere, and a good many other spheres as well if time and opportunity served. But we must carry our physic with us, for physic, in the widest sense of that word, is destined to play a much more conspicuous and important part in the life of the future than it has done in that of the past.

In very remote ages, and sometimes still in outlying country districts, nature makes the roads, and man mends them; and very bad roads they generally are. Similarly in past times the world has been content that nature should make men and women; and that man, that is the doctor, should mend them when they fell into disrepair. Nowadays we make the highways for ourselves instead of leaving them to nature, with the result that we travel on sound, hard, dry roads, and do not need to spend an annual fortune in rendering them passable. On the same lines doctors of original minds purpose taking a share in the shaping and making of the man of the future. The mere mending of him when he is out of repair is by no means a sufficient function for the ambition of the scientific medical intelligence.

But it is one thing to see a great possible conquest: it is quite another to achieve it and to make it permanent. If medicine is to expand with our growing empire it will have to lay aside a good many of its impedimenta, and to arm itself anew. At the present time British medicine is marked by two striking defects: it has little order and less simplicity. If we take that part of it which deals with the various methods of healing—with treatment, the whole business is in a state of indescribable confusion. Doctors with unusually clear heads and honest consciences, it is true, have very definite and mostly simple and successful methods of restoring their patients to health. But they owe that almost entirely to themselves, and very little, if at all, to any accepted philosophy of medicine, or to the way in which the medical art is taught at the schools. Details, details, and yet more details, are the beginning and the ending of nearly every medical teacher's gospel. Drugs, drugs, and yet more drugs are the feeble, untried, and often useless weapons with which he combats the maladies and the disabilities of mankind. A bewildered student of Materia Medica may well ask where are the principles that are to guide and govern us in the every-day practice of our art?

It is not merely in the largely unscientific use of drugs that British medicine is in so chaotic and unintelligent a condition: the very nature of disease itself seems to be as great a mystery to many medical minds as it was to the ancient Britons before the advent of Cæsar. Young men are taught to recognize diseases, not to understand disease. Intelligent science is always trying to find the one in the many; and to reduce the many under the headship of the one; to classify, in short, and to separate similars from dissimilars, and identicals from opposites. If an average medical man were asked to give a perfectly simple definition of disease which an intelligent child could understand, he would signally fail. But that is a thing which medical men should feel anything but proud of. Disease, in its essence, does not consist of tens of thousands of different pathological activities, each more mysterious than another, but of a strictly limited number of definable processes, which manifest themselves in the various organs of the body and under a variety of circumstances. Almost every form of disease may be classed under one of two heads—inflammation and degeneration. Nearly every disease, for example, is occasioned either by a super-normal quantity or quality of blood, or by a sub-normal quantity or quality of blood. But the very fact that almost all diseases can be classed under two general heads shows that there are dividing lines of great definiteness which separate diseases one from another, and which make classification and simplicity at least possible, if not easy.

Treatment, like diseases, may be generalised and classified. For example, there is a whole range of pathological processes in which the blood, which is the life, is poorer in quality and less in quantity than it ought to be. There is another class in which the blood is either in excess, or in a state of excessive activity, or is injuriously altered by foreign additions. Every medical man should have these two generalisations as constantly and practically present in his mind as the musician has his trained musical sense. For the state of the blood is usually the basis and beginning of all morbid activity; and the strongest battalions of treatment should be led against it. In other words, treatment should always be fundamental, whether it be symptomatic or not. The skilful physician will have regard to the symptoms in due measure as well as to the fundamental character of the diseased process. But he who treats symptoms only, whilst failing to understand and to manage the actual disease, is like a rider on a runaway horse, who instead of firmly reining in his steed or guiding it wisely until it is "winded" and stops, busies himself with flicking the flies off its flanks. If such a rider does not break both his own and his horse's neck, he has to thank his good fortune and not his skill. The one conspicuous defect in the teaching of modern medicine is the want of clear insistence on broad, general principles. We are well aware of the importance of details; but if the medical profession as a whole is to be rationally and practically skilful, it must have a perfect, easy, and constant familiarity with the leading principles of diseased processes, and with the main lines of genuinely scientific treatment.

As the British people spreads over the globe, carrying with it, not the details of English life and manners, but the general principles of practical common sense, indomitable will, and ready adaptability to every variety cf circumstance, so must the British doctor first be thoroughly grounded in the great facts of health and disease, and in the few proved and accepted principles of the therapeutic management of the sick. These broad generalisations constitute the bony skeleton of his science and art; and just as a mollusc is in every way a lower and less worthy animal than a vertebrate, though he be a hundred times the size, so the medical man whose science and art consist of a mere disordered mass of details, however large the mass may be, is altogether inferior for every purpose to the man who has arranged his knowledge and practice on sound fundamental principles of physiology and of the known action of those remedial agents which long experience has tried and proved. If the greater part of the world is to be covered by British men, it ought also to be covered by British medicine. We all have a right to ask medical teachers that they shall train and equip a class of men who, for breadth of knowledge, soundness of judgment, and practical capacity for applying the great principles of the healing art, shall be worthy of their fellow countrymen, with whom they go forth into unknown lands, and of the noble home of freedom and intellectual strength which they leave behind them.

